# Gene expression profiling of flax (*Linum usitatissimum* L.) under edaphic stress

**DOI:** 10.1186/s12870-016-0927-9

**Published:** 2016-11-16

**Authors:** Alexey A. Dmitriev, Anna V. Kudryavtseva, George S. Krasnov, Nadezhda V. Koroban, Anna S. Speranskaya, Anastasia A. Krinitsina, Maxim S. Belenikin, Anastasiya V. Snezhkina, Asiya F. Sadritdinova, Natalya V. Kishlyan, Tatiana A. Rozhmina, Olga Yu. Yurkevich, Olga V. Muravenko, Nadezhda L. Bolsheva, Nataliya V. Melnikova

**Affiliations:** 1 Engelhardt Institute of Molecular Biology, Russian Academy of Sciences, Moscow, Russia; 2 Faculty of Biology, Lomonosov Moscow State University, Moscow, Russia; 3 All-Russian Research Institute for Flax, Torzhok, Russia

**Keywords:** *Linum usitatissimum*, Flax, Edaphic stress, Phosphate deficiency, Excess nutrition, LIS-1, WRKY, JAZ, qPCR, High-throughput sequencing

## Abstract

**Background:**

Cultivated flax (*Linum usitatissimum* L.) is widely used for production of textile, food, chemical and pharmaceutical products. However, various stresses decrease flax production. Search for genes, which are involved in stress response, is necessary for breeding of adaptive cultivars. Imbalanced concentration of nutrient elements in soil decrease flax yields and also results in heritable changes in some flax lines. The appearance of Linum Insertion Sequence 1 (LIS-1) is the most studied modification. However, LIS-1 function is still unclear.

**Results:**

High-throughput sequencing of transcriptome of flax plants grown under normal (N), phosphate deficient (P), and nutrient excess (NPK) conditions was carried out using Illumina platform. The assembly of transcriptome was performed, and a total of 34924, 33797, and 33698 unique transcripts for N, P, and NPK sequencing libraries were identified, respectively. We have not revealed any LIS-1 derived mRNA in our sequencing data. The analysis of high-throughput sequencing data allowed us to identify genes with potentially differential expression under imbalanced nutrition. For further investigation with qPCR, 15 genes were chosen and their expression levels were evaluated in the extended sampling of 31 flax plants. Significant expression alterations were revealed for genes encoding WRKY and JAZ protein families under P and NPK conditions. Moreover, the alterations of WRKY family genes differed depending on LIS-1 presence in flax plant genome. Besides, we revealed slight and LIS-1 independent mRNA level changes of *KRP2* and *ING1* genes, which are adjacent to LIS-1, under nutrition stress.

**Conclusions:**

Differentially expressed genes were identified in flax plants, which were grown under phosphate deficiency and excess nutrition, on the basis of high-throughput sequencing and qPCR data. We showed that WRKY and JAS gene families participate in flax response to imbalanced nutrient content in soil. Besides, we have not identified any mRNA, which could be derived from LIS-1, in our transcriptome sequencing data. Expression of LIS-1 flanking genes, *ING1* and *KRP2*, was suggested not to be nutrient stress-induced. Obtained results provide new insights into edaphic stress response in flax and the role of LIS-1 in these process.

**Electronic supplementary material:**

The online version of this article (doi:10.1186/s12870-016-0927-9) contains supplementary material, which is available to authorized users.

## Background

Cultivated flax (*Linum usitatissimum* L.) is widely used for production of textile, chemicals, food, and pharmaceutical products. However, various biotic and abiotic stresses decrease flax oil and fiber yield. Therefore, cultivation of resistant to unfavorable environment *L. usitatissimum* varieties is required for obtaining of high and stable yield. Understanding of mechanisms of flax stress resistance is necessary for breeding of cultivars, which are tolerant to stress conditions. Search for genes, which are involved in stress response, is important for progress in genetics of tolerance and genomics-assisted breeding of flax. Edaphic stresses, including deficient or excessive nutrition, significantly limit the production of flax [1], and is of particular interest for investigation. Moreover, imbalanced concentration of nutrient elements in soil result in heritable phenotype and genotype changes in some flax lines, which were called plastic [2–9]. Offspring of plastic line, which stably inherited the changes, were termed genotrophs [7]. The most famous flax plastic line is ‘Stormont cirrus’ and the most studied genetic modification is Linum Insertion Sequence 1 (LIS-1). LIS-1 is a 5.7-kb sequence that appear into a particular site of flax genome under nutrient deficiency, however, LIS-1 function is still unclear [2–4, 10, 11].

In the present work, we investigated the impact of soil nutrient stress on flax plants and compared the responses to nutrient deficiency or excess in line ‘Stormont cirrus’ with and without LIS-1 and non-plastic cultivar ‘Bethune’. High-throughput sequencing and quantitative RCR (qPCR) were used for gene expression evaluation in flax plants and allowed us to identify genes, which are likely involved in response to unfavorable nutrient content in soil.

## Methods

### Plant material

*L. usitatissimum* plants of cultivar ‘Bethune’ and line ‘Stormont Cirrus’ with and without LIS-1 were grown under normal (N), phosphate deficiency (P) and excess nutrition (NPK) conditions as described earlier [11]. Plant material was obtained from nine plants of ‘Bethune’ (three plants were grown under N conditions, 3 – under P, and 3 – under NPK), ten plants of ‘Stormont Cirrus’ with stable presence of LIS-1 (3 – N, 3 – P, and 4 – NPK), and 12 plants of ‘Stormont Cirrus’ with stable absence of LIS-1 (4 – N, 4 – P, 4 – NPK). Upper leaves from individual plants after 6 weeks of growth were collected and immediately frozen in liquid nitrogen. Plant samples were stored at −70 °C. Total DNA was isolated using chloroform:isoamyl alcohol extraction as described earlier [12]. Total RNA was extracted using RNA MicroPrep kit (Zymo Research, USA). The LIS-1 status of each individual plant was tested by PCR with primers of Chen et al. [3] and agarose gel electrophoresis as described earlier [11].

### Transcriptome sequencing

CDNA library preparation was performed using TruSeq RNA SamplePrep (Illumina, USA). Poly-A containing mRNA fraction was isolated using poly-T oligoattached magnetic beads. Pooled samples were used to prepare three libraries for line ‘Stormont Cirrus’ grown under N, P, and NPK conditions. Library quality was evaluated using Agilent 2100 Bioanalyzer (Agilent Technologies, USA), then libraries were sequenced on Illumina GAIIx platform. Raw reads were trimmed and filtered with trimmomatic [13]. The assembly of transcriptome was performed using SOAPdenovo-Trans transcriptome assembler. Assembled transcripts were mapped to *L. usitatissimum* ‘reference’ transcriptome database of JGI Genome portal with blastn (PhytozomeV11: Lusitatissimum_200_v1.0.transcript.fa.gz; [14]). If several assembled transcripts were mapped to one ‘reference’ transcript, then we merged them into one transcript and filled gaps from the ‘reference’ sequence. Next we transferred annotations from ‘reference’ dataset to the assembled transcripts. For the further analysis, only mapped transcripts were used that made assembly more reliable.

To assess differential expression, reads were aligned to the assembled transcripts with bowtie2 [15] and read count per transcript values were calculated with rsem [16]. Library normalization was performed with total number of reads. The comparison of mRNA levels in N, P, and NPK libraries was carried out using *fold change* (normalized read count in NPK or P/normalized read count in N) and *p-value* (*χ* ^*2*^ test) parameters [17].

Gene set enrichment analysis with Gene Ontology data was performed using Goseq for genes with the highest fold changes (http://bioconductor.org/packages/release/bioc/html/goseq.html).

For identification of mRNAs derived from LIS-1 or its 5′ and 3′ flanking sequences, obtained Illumina transcriptomic reads were aligned to GenBank accessions AF104351.1, AF300798.1, and AF300797.1 using blastn [18].

### QPCR analysis

PCR reactions were performed using a 7500 Real-Time PCR System (Applied Biosystems, USA) in 20 μl reaction mix containing 1× 2-FRT PCR mix (Amplisens, Russia), 250 nM of dNTPs mix (Fermentas, Lithuania), 350 nM of forward and reverse primers, 2 U of TaqF polymerase (Amplisens), 1× EvaGreen (Biotium, USA), and cDNA. The following amplification program was used: 95 °C for 15 min; 50 cycles of 95 °C for 15 s, 60–62 °C for 60 s. In total, nine flax plants of cultivar ‘Bethune’, ten plants of line ‘Stormont Cirrus’ with LIS-1 and 12 plants without LIS-1, which were grown under N (ten plants), P (10 plants), and NPK (11 plants) conditions, were studied. Expression of 15 genes was evaluated with primers, which were selected using NCBI Primer Blast [19] preferably with amplicon spanning exon-exon junction (see Additional file 1: Table S1). For qPCR data analysis, reference genes *ETIF3H* and *ETIF3E* [20] were used. All calculations were performed using the program ATG (**A**nalysis of **T**ranscription of **G**enes) [21]. The *ΔC*_*t*_^*eff*^ values, which are directly proportional to the expression levels, were calculated according to the formula [11]:

$$ \mathit{\mathsf{\varDelta}}\left({C}_t^{eff}\right)={\left({C}_t^{eff}\right)}^{\mathit{\mathsf{reference}}\ \mathit{\mathsf{gene}}}\hbox{-} {\left({C}_t^{eff}\right)}^{\mathit{\mathsf{target}}\ \mathit{\mathsf{gene}}} $$

$$ {C}_t^{eff}={C}_t \times lo{g}_2\left(1+E\right) $$

where *Ct*, replicate-averaged threshold cycle; *E*, efficiency of reaction for each pair of primers. Each qPCR reaction was repeated three times. Amplification efficiency was 95 % or higher, threshold cycle varied from 23 to 34 for all primer pairs (Additional file 1: Table S1).

Kruskal–Wallis and Mann-Whitney tests were used for assessment of statistical significance of revealed expression alterations. *P* ≤ 0.05 were considered statistically significant.

## Results

### High-throughput sequencing of N, P, and NPK flax plant libraries

Three transcriptome libraries were constructed from flax plants grown under normal (N), phosphate deficient (P), and nutrient excess (NPK) conditions. High-throughput sequencing using GAIIx Illumina platform generated a total 10.6, 13.6, and 18.6 million reads (75 nucleotides in length) for N, P, and NPK conditions, respectively (Sequence Read Archive – SRP083007). All three libraries demonstrated very similar statistics: GC content was equal to 47–48 %, average quality per read was about 38, PCR sequence duplication level varied from 15 to 17 %. After mapping to the reference *L. usitatissimum* transcriptome (JGI Genome portal), joining hits and filling gaps, 34924, 33797, and 33698 unique transcripts were identified for N, P, and NPK libraries, respectively. The maximum transcript length was 14619 nucleotides.

### Gene expression analysis on the basis of high-throughput sequencing data

**Fig. 1:**
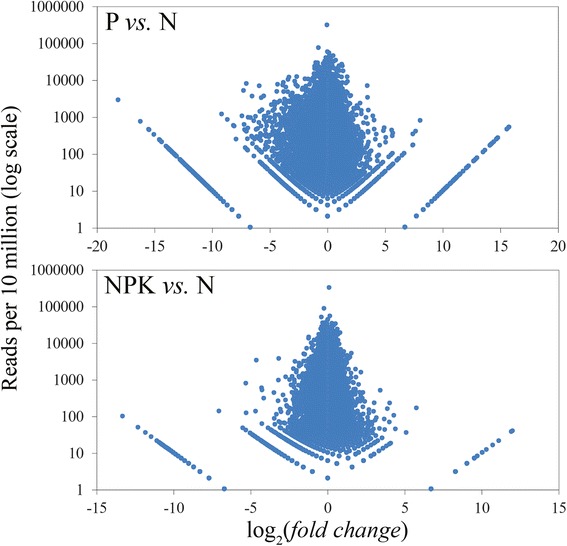
Relative and absolute expression levels of genes in plants grown under phosphate deficiency (P *vs.* N) or excess nutrition (NPK *vs.* N). High-throughput sequencing data. Each point represents the data for one gene. Y-axis – the sum of normalized reads in normal (N) and nutrient stress (P or NPK) conditions

The number of reads aligned to each assembled transcript was determined and normalized per 10 million reads. For the visualization of expression analysis results, the diagrams were constructed (Fig. 1). Gene Ontology enrichment analysis was performed for top 30 differentially expressed genes. Both under P and NPK conditions, expression of the majority of genes from the top 30 was decreased. Growing of flax plants under phosphate deficiency resulted mainly in the decrease of expression of transcription factors (*p* = 2.4*10^−7^). Thus, transcription factors could play an important role in flax adaptation to nutrition stress that is in concordance with data on the role of transcription factors in plant tolerance to abiotic stresses [22]. Under P conditions, expression alterations of genes, which participate in histone acetyltransferase activity (*p* = 2.9*10^−5^), sequence-specific DNA binding (*p* = 8.0*10^−5^), asparagine biosynthetic process (*p* = 2.1*10^−3^), protein binding process (*p* = 4.8*10^−3^), zinc ion binding (*p* = 0.03), and methyltransferase activity (*p* = 0.07) was also revealed. Under NPK conditions, differentially expression was observed for genes, which participate in histone acetyltransferase activity (*p* = 4.6*10^−4^), iron ion binding (*p* = 4.6*10^−4^), transcription cofactor activity (*p* = 4.6*10^−4^), oxidoreductase activity (*p* = 2.6*10^−3^), protein binding (*p* = 3.4*10^−3^), ion channel activity (*p* = 0.02), *etc*.

### LIS-1 derived RNAs

**Fig. 2:**
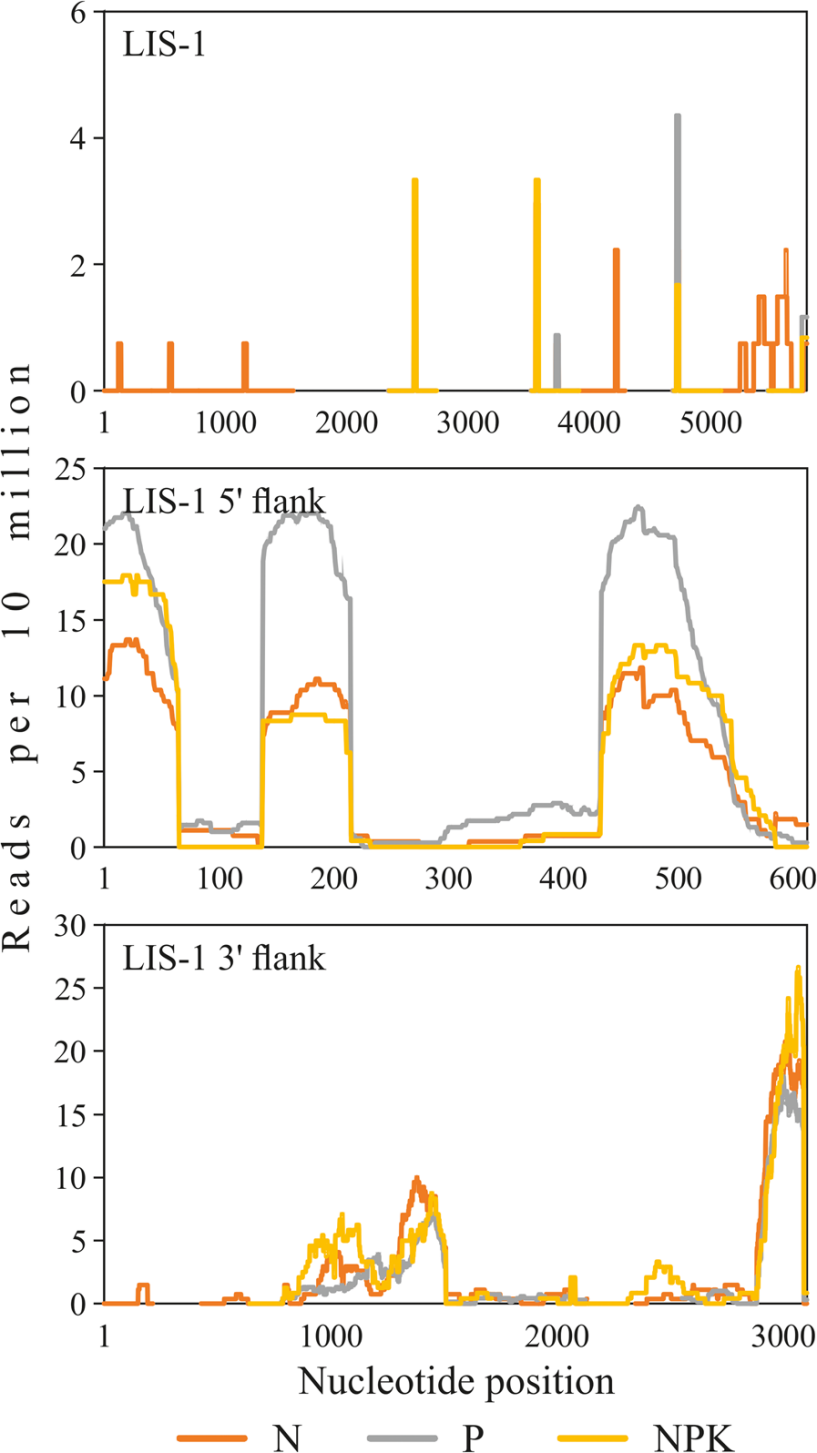
The coverage of LIS-1, LIS-1 5′ and 3′ flanking sequences. High-throughput sequencing data. N – normal conditions, P – phosphate deficiency, NPK – excess nutrition. GenBank accessions: LIS-1 – AF104351.1, LIS-1 5′ flank – AF300797.1, LIS-1 3′ flank – AF300798.1

For identification of mRNAs derived from LIS-1, its 5′ and 3′ flanking sequences, obtained reads were aligned to GenBank accession AF104351.1, AF300797.1, and AF300798.1, respectively. For LIS-1 5′ and 3′ flanking sequences, coincidence was revealed for regions, which are corresponded to exons of Inhibitor of growth 1 (*ING1*) and Kip-related cyclin-dependent kinase inhibitor 2 (*KRP2*) genes (Fig. 2). Expression analysis on the basis of our high-throughput sequencing data revealed no difference in expression level of *KRP2* gene under N, P, and NPK conditions. A slight increase of expression was observed for *ING1* gene under P conditions. For LIS-1, we identified only few read k-mers, which coincided with short regions of the sequence. Moreover, matched k-mers were identified in both sample with LIS-1 and samples without LIS-1. Therefore, we concluded that no reads were derived from LIS-1.

### QPCR analysis of gene expression in flax plants grown under imbalanced nutrition

We used high-throughput sequencing data for three pooled plant samples grown under different nutrition conditions for preliminary evaluation of gene expression. For reliable evaluation of expression alterations, we performed qPCR analysis in the extended sampling (ten flax plants grown under N, 11 – under P, and 10 – under NPK conditions). On the basis of high-throughput sequencing data, 20 genes with up- or down-regulation under nutrition stress were chosen. Twenty pairs of primers for qPCR were designed and tested, but only 13 of them showed high efficiency and specificity. QPCR analysis was performed for plants of line ‘Stormont cirrus’ with and without LIS-1 and cultivar ‘Bethune’ to evaluate the expression of genes encoding the following proteins: WRKY DNA-binding protein 70 (WRKY70); WRKY DNA-binding protein 33 (WRKY33); WRKY DNA-binding protein 40 (WRKY40); JAZ8/TIFY5A jasmonate-zim-domain protein 8 (JAZ8); JAS1, JAZ10, TIFY9 jasmonate-zim-domain protein 10 (JAZ10); Putative nuclease HARBI1 (HARBI1); S-adenosyl-L-methionine-dependent methyltransferase (SAM MTase); Neutral invertase (A/N-Invs); CCR4-associated factor 1 homolog 11 (CAF1-11); BTB and TAZ domain protein 4 (BT4); MLP-like protein 423 (MLP423); Ethylene-responsive transcription factor ERF (ERF); Myb domain protein 41 (MYB41). Besides, expression of *ING1* and *KRP2* genes, which are located in LIS-1 flanking sequences, was evaluated.

**Fig. 3:**
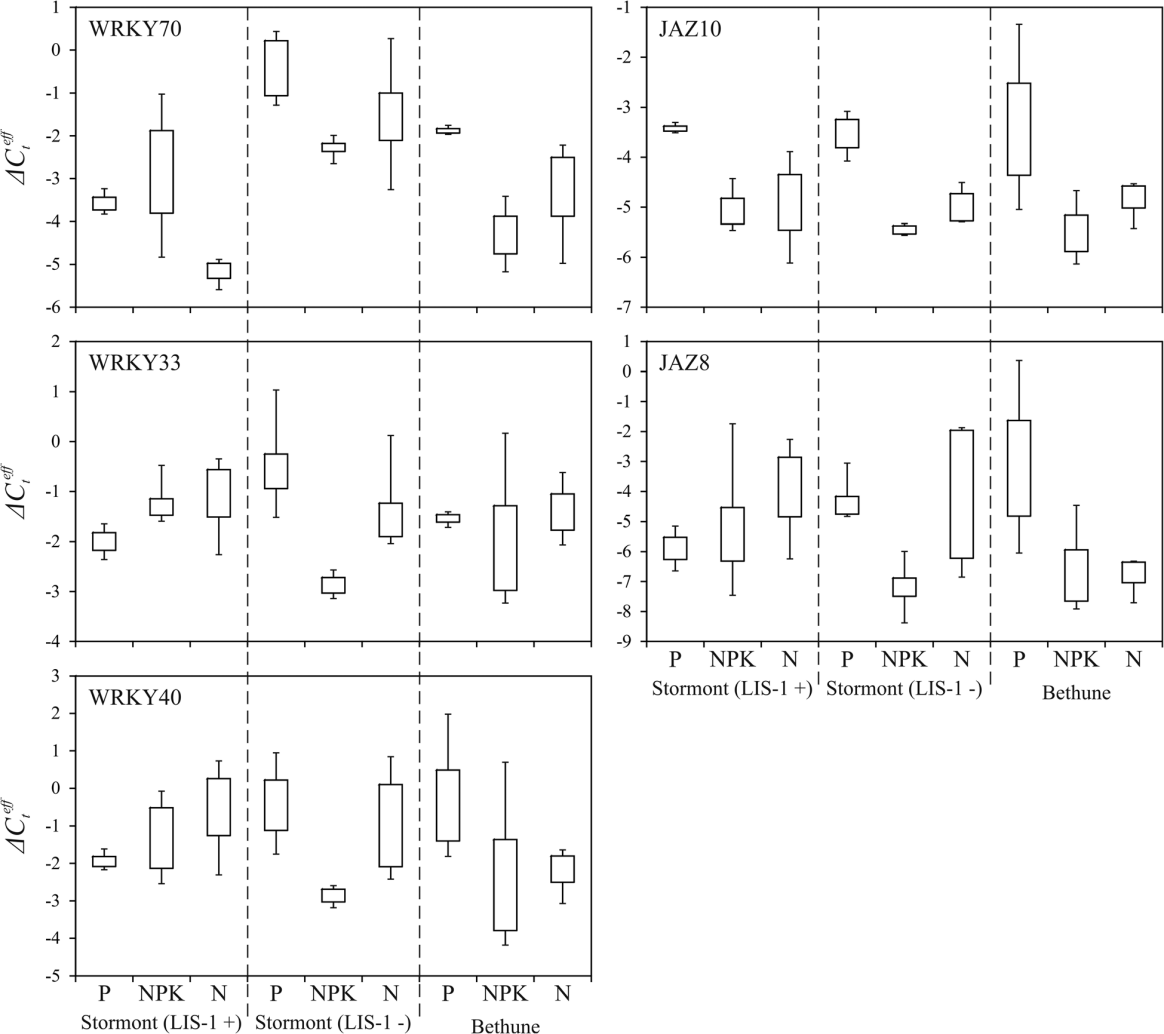
Expression of five genes in plants of cultivar ‘Bethune’ and line ‘Stormont Cirrus’ with (+) and without (-) LIS-1 grown under phosphate deficiency (P), excess nutrition (NPK), or normal (N) conditions. QPCR data. Rectangles correspond to the ranges containing 50 % of the values (between the 25^th^ and 75^th^ percentiles); the bars are the maximum and minimum Δ*C*_*t*_^*eff*^ values

Significant expression alterations were revealed for genes encoding WRKY DNA-binding protein family and genes encoding JAZ jasmonate-zim-domain protein family in ‘Stormont cirrus’ plants with and without LIS-1 as well as in ‘Bethune’ plants grown under different nutrient conditions (Fig. 3).

For JAZ gene family, the reaction on different nutrition conditions was similar in the majority of groups of samples – expression was increased under P compared to NPK conditions. Median of $\varDelta {C}_t^{eff}$ for *JAZ10* was −3.4 under P and −5.1 under NPK in ‘Stormont cirrus’ with LIS-1; −3.5 under P and −5.5 under NPK in ‘Stormont cirrus’ without LIS-1; −3.7 under P and −5.6 under NPK in ‘Bethune’. Median of $\varDelta {C}_t^{eff}$ for *JAZ8* was −5.9 under P and −5.7 under NPK in ‘Stormont cirrus’ with LIS-1; −4.6 under P and −7.2 under NPK in ‘Stormont cirrus’ without LIS-1; −3.7 under P and −7.4 under NPK in ‘Bethune’. JAZ family proteins are repressors of jasmonic acid, which regulates many aspects of plant growth, development and stress responses [23]. Our results suggest that JAZ family proteins are involved in response to deficient and excessive nutrition in flax.

For WRKY gene family, expression was also increased under P compared to NPK conditions, but only in genotypes without LIS-1. Median of $\varDelta {C}_t^{eff}$ for *WRKY70* was −0.4 under P and −2.3 under NPK in ‘Stormont cirrus’ without LIS-1; −1.9 under P and −4.3 under NPK in ‘Bethune’. Median of $\varDelta {C}_t^{eff}$ for *WRKY33* was −0.7 under P and −2.9 under NPK in ‘Stormont cirrus’ without LIS-1; −1.5 under P and −2.7 under NPK in ‘Bethune’. Median of $\varDelta {C}_t^{eff}$ for *WRKY40* was −0.5 under P and −2.9 under NPK in ‘Stormont cirrus’ without LIS-1; −1.0 under P and −3.4 under NPK in ‘Bethune’. For ‘Stormont cirrus’ with LIS-1 the trend was reverse: median of $\varDelta {C}_t^{eff}$ for *WRKY70* was −3.6 under P and −2.8 under NPK; for *WRKY33 –* –2.0 under P and −1.4 under NPK; for *WRKY40 ﻿–* −2.0 under P and −1.3 under NPK. Proteins of WRKY family are transcription factors, which are involved in many processes in plants including stress response [24]. On the basis of our data, we can speculate that the genes of this family participate in flax response to imbalanced nutrition. It is worth noting that expression alterations of WRKY family genes differed depending on LIS-1 presence in flax plant genome.

For the other examined genes, statistically significant expression alterations were revealed only for particular groups of samples (Additional file 2: Figure S1). Expression was altered in ‘Stormont cirrus’ with LIS-1 for genes encoding Putative nuclease HARBI1 (median of $\varDelta {C}_t^{eff}$ was −5.2 under P, −3.8 under NPK, and −2.8 under N), MLP423 (−4.2 under P, −2.9 under NPK, and −4.8 under N), ING1 (−3.4 under P and −2.4 under NPK), A/N-Invs (−1.5 under P and 0.2 under NPK), BT4 (−4.2 under P and −3.0 under NPK); in ‘Stormont cirrus’ without LIS-1 – for genes encoding ING1 (−4.2 under P and −3.2 under N), BT4 (−4.6 under P and −3.1 under N), KRP2 (−1.8 under P, −2.4 under NPK, and −2.0 under N), and ERF (−3.4 under P, −7.2 under NPK, and −5.7 under N); in ‘Bethune’ – only for MYB41 (−1.8 under P, −3.7 under NPK, and −2.6 under N). For genes encoding CAF1-11 and SAM MTase, significant expression alterations were not revealed. Thus, the majority of expression alterations of listed above genes under imbalanced nutrition were observed for plastic line ‘Stormont cirrus’, while in non-plastic cultivar ‘Bethune’ only one gene was differentially expressed.

## Discussion

### The role of LIS-1 in response to imbalanced nutrition

High-throughput sequencing of three cDNA libraries, which were prepared from flax plants grown under different nutrition conditions, was performed. Transcriptome assembly enabled identification of 34924, 33797, and 33698 unique transcripts for N, P, and NPK libraries, respectively. For recognition of LIS-1 derived reads, we chose Blast due to its tolerance to mismatches and long insertions/deletions since one cannot exclude high polymorphism of LIS-1 between genotypes. As a result, we have not revealed any LIS-1 derived mRNA in our sequencing data. Earlier, it was reported that LIS-1 sequence did not contain large open reading frames [4]. In our previous study, we have not identified any LIS-1 transcribed microRNA as well [11]. In the present work, we revealed statistically significant expression alterations of *KRP2* and *ING1* genes, which are adjacent to LIS-1, under imbalanced nutrition conditions. However, the degree of changes was not high and correlation between LIS-1 status and expression alterations was not observed. For *ING1* gene, a slight increase of expression was revealed under P conditions in all groups of samples: ‘Stormont cirrus’ with LIS-1 – median $\varDelta {C}_t^{eff}$: −3.4 in P and −2.8 in N; ‘Stormont cirrus’ without LIS-1 – median $\varDelta {C}_t^{eff}$: −4.2 in P and −3.2 in N; ‘Bethune’ – median $\varDelta {C}_t^{eff}$: −3.8 in P and −2.9 in N. Expression level of *KRP2* gene varied slightly under different nutrition conditions (median $\varDelta {C}_t^{eff}$ was in the range from 1.4 to 2.4 in all groups of samples). Therefore, we can conclude that LIS-1 presence most likely does not result in expression alterations of its flanking genes under nutrient stress.

### Evaluation of the gene expression alterations under P and NPK condition

For our study, we used seeds of plastic line ‘Stormont cirrus’ obtained from plants with stable status of LIS-1: with LIS-1 and without LIS-1. Also, we used non-plastic cultivar ‘Bethune’ in which LIS-1 never occurs. Flax plants were grown under N, P, and NPK conditions and qPCR analysis was performed to assess the expression of genes, which were chosen on the basis of high-throughput sequencing data, in extended sampling of 31 plants. Alike expression alterations were revealed for studied genes of JAZ family (*JAZ8* and *JAZ10*) under N, P, and NPK conditions for the majority of groups: expression increased under P conditions and decreased under NPK compared to N (only for ‘Stormont cirrus’ with LIS-1, expression of *JAZ8* was almost the same under P and NPK conditions). JAZ family proteins are repressors of phytohormone jasmonates, which play important role in plant growth, development and stress tolerance [23, 25–27]. Also, the interaction of ubiquitination process and jasmonate signaling was revealed [28]. However, little is known about the role of JAZ proteins in response to nutrient stress. Differential expression of TIFY family genes under phosphate deficiency was revealed in common bean after 25 days of growth: some of TIFY family genes were induced and others were repressed. Participation of TIFY family genes in *Phaseolus vulgaris* response and adaptation to P-starvation was assumed [29]. The involvement of JAZ proteins in nutrient deficiency response was revealed in rice and chickpea: differential expression was shown under macro and micronutrients deficiency; most of chickpea and rice *JAZ* genes were up-regulated after 7 days and down-regulated after 15 days of phosphate deficiency [30]. In the present work in flax plants after 6 weeks of growth under phosphate deficiency, *JAZ* genes were up-regulated, while under excess nutrition, *JAZ* genes were down-regulated. Obtained results indicate the involvement of JAZ proteins in flax response to imbalanced nutrition. Our data make a contribution to the knowledge on the role of JAZ proteins in abiotic stress response in plants.

Significant expression alterations in flax plants grown under imbalanced nutrition were revealed for genes of WRKY family (*WRKY33*, *WRKY40*, and *WRKY70*). WRKY gene family, one of the largest families of transcription factors in plants, participates in diverse biological processes, including response to biotic and abiotic stresses [31]. Numerous studies on WRKY protein role were performed for different plant species [31, 32]. Deficiency of nutrition elements or their imbalance in soil is a serious problem for agricultural production, so, plant response to this stress is intensively studied. It was shown that WRKY family genes are involved in tolerance to phosphate starvation in rice: overexpression of *WRKY74* gene increase tolerance to phosphate starvation, while lines with low *WRKY74* level were sensitive to this stress [33]. In *Arabidopsis*, WRKY42 regulates phosphate homeostasis: *WRKY42* overexpressing lines were more sensitive to phosphate deficiency [34]. However, overexpression of *WRKY45* in *Arabidopsis* increased phosphate uptake [35]. For flax, the involvement of WRKY family genes only in saline-alkaline stress response was known [36, 37]. In the present work, we revealed significant expression decrease of *WRKY33*, *WRKY40*, and *WRKY70* under NPK compared to P conditions for flax genotypes without LIS-1 (‘Stormont cirrus’ without LIS-1 and ‘Bethune’). However, for ‘Stormont cirrus’ with LIS-1, expression increase or retention of studied WRKY family genes was found under NPK compared to P conditions. It is known that flax small genotrophs with LIS-1 are more adaptive to nutrient deficiency in comparison with lines without LIS-1 [2]. In the present study, we showed that in more adaptive to P conditions ‘Stormont cirrus’ genotypes (‘Stormont cirrus’ with LIS-1), expression of WRKY family genes was up-regulated in contrast to less adaptive ones (‘Stormont cirrus’ without LIS-1). In ‘Bethune’ cultivar, expression alterations of WRKY family genes were similar to ‘Stormont cirrus’ without LIS-1. We assumed that WRKY family genes are involved in nutrition stress response in flax and their overexpression is a feature of less adaptive to phosphate deficiency lines and cultivars.

## Conclusions

Search for genes, which are involved in flax response to imbalanced nutrition, was performed. Differentially expressed genes were identified in flax plants, which were grown under phosphate deficiency and excess nutrition, on the basis of high-throughput sequencing and qPCR data. Expression of WRKY and JAS gene families altered in all examined groups of flax plants that allowed us to assume the important role of these genes in flax response to nutrition stress. Moreover, the expression level of WRKY family genes was different in responsive and adaptive to phosphate deficiency genotypes, which differed by LIS-1 presence. Besides, we have not identified any mRNA, which could be derived from LIS-1, in our transcriptome sequencing data. Expression alterations of LIS-1 flanking genes, *ING1* and *KRP2*, were slight and did not correlate with LIS-1 presence in flax plant genome pointing that these genes are not nutrient stress-induced in flax. These data provide new insights into edaphic stress response in flax and the role of LIS-1 in this process.

## Additional files

Additional file 1: Table S1. Primer sequences, amplification efficiencies and threshold cycles (Ct) for genes examined in the study. (DOCX 17 kb)
Additional file 2: Figure S1. Expression of ten genes in plants of cultivar ‘Bethune’ and line ‘Stormont Cirrus’ with (+) and without (-) LIS-1 grown under phosphate deficiency (P), excess nutrition (NPK), or normal (N) conditions. QPCR data. Rectangles correspond to the ranges containing 50 % of the values (between the 25th and 75th percentiles); the bars are the maximum and minimum Δ*C*_*t*_^*eff*^ values. (TIF 873 kb)
