# Supplementary material for: Gene expression profiling of flax (Linum usitatissimum L.) under edaphic stress
Source: BMC Plant Biol. 2016 Nov 16;16(Suppl 3):139–46. doi: 10.1186/s12870-016-0927-9 (PMC5123303; doi:10.1186/s12870-016-0927-9)
Supplement: Additional file 1: Table S1. — Primer sequences, amplification efficiencies and threshold cycles (Ct) for genes examined in the study. (DOCX 17 kb) [file 12870_2016_927_MOESM1_ESM.docx]

**Table S1.** Primer sequences, amplification efficiencies and threshold cycles (Ct) for genes examined in the study.

| **Gene** | **Primer sequence** | **Amplification**  **efficiency** | **Ct** |
| --- | --- | --- | --- |
| *WRKY70* | F: GCTCCTCCACCAAGGATGAC | 97 | 24-33 |
|  | R: GTACTTCCTCCACTGGTGCC |  |  |
| *WRKY33* | F: TGACGGATACAGGTGGAGGA | 95 | 24-31 |
|  | R: CCTGACAGGACATCCTACGAA |  |  |
| *WRKY40* | F: GGCAGAAGGTGACCAGAGAT | 96 | 23-30 |
|  | R: CCACTACCGATTGGTCCTCC |  |  |
| *JAZ10* | F: AATGGCCTTCCATCCTCACC | 97 | 28-32 |
|  | R: AACCTCTGCAACGACTTCCG |  |  |
| *JAZ8* | F: ATTCTACGAGGGGAGGTGCT | 95 | 26-34 |
|  | R: GTCCTCTGCTCTTGAACGCT |  |  |
| *HARBI1* | F: ATCAGCGTGGCTGCTTACTT | 95 | 24-33 |
|  | R: GAACCAGGCCAACCGATACA |  |  |
| *SAM MTase* | F: CTGGCGAGACAAGTTACGCT | 95 | 26-31 |
|  | R: GGAAGGTTGAGCTGCAGAGT |  |  |
| *A/N-Invs* | F: GGAAGGTTGAGCTGCAGAGT | 96 | 23-30 |
|  | R: GCTGGCATTAACCCTTGTCC |  |  |
| *CAF1-11* | F: TCAGGGGATCGACTTCGCTA | 97 | 25-34 |
|  | R: GCGTCGTTGCATACAAGTCC |  |  |
| *BT4* | F: CGCCGTTCAAGTCTTCATCA | 95 | 25-34 |
|  | R: AGATTCGTTTCAGCTCCGGC |  |  |
| *MLP423* | F: CGCCGCTACAACTGTTTTCC | 96 | 19-33 |
|  | R: TGACCGAAAGTGATGGTGCG |  |  |
| *ERF* | F: GACCGCATTTGGTTGGGTTC | 96 | 23-32 |
|  | R: GTGGGTCCACGTAAGCAGAA |  |  |
| *MYB41* | F: CCTACATCACTCGCTACGGC | 98 | 25-31 |
|  | R: TCCCTCGTTTGAGATCCGGT |  |  |
| *ING1* | F: ACTCGTCAAGGAAAGACATTGGT | 96 | 26-31 |
|  | R: AGGGGGCGGATAACAGAAGA |  |  |
| *KRP2* | F: TGGTTTGAAACTCGTCAAGGA | 97 | 25-30 |
|  | R: CGGGAAGGGTCATCACGTT |  |  |
| *ETIF3E** | F: TTACTGTCGCATCCATCAGC | 99 | 23-28 |
|  | R: GGAGTTGCGGATGAGGTTTA |  |  |
| *ETIF3H** | F: CAGCGTGCTTGAAGTAACCA | 98 | 24-29 |
|  | R: AACCTCCCTCAAGCATCTCA |  |  |

*Note*: * – Primer sequences are from Huis et al. article [[1](#_ENREF_1)].

**References**

1. Huis R, Hawkins S, Neutelings G. Selection of reference genes for quantitative gene expression normalization in flax (Linum usitatissimum L.). BMC plant biology. 2010;10:71.
