# Supplementary figures and images for: Gene expression profiling of flax (Linum usitatissimum L.) under edaphic stress
Source: BMC Plant Biol. 2016 Nov 16;16(Suppl 3):139–46. doi: 10.1186/s12870-016-0927-9 (PMC5123303; doi:10.1186/s12870-016-0927-9)

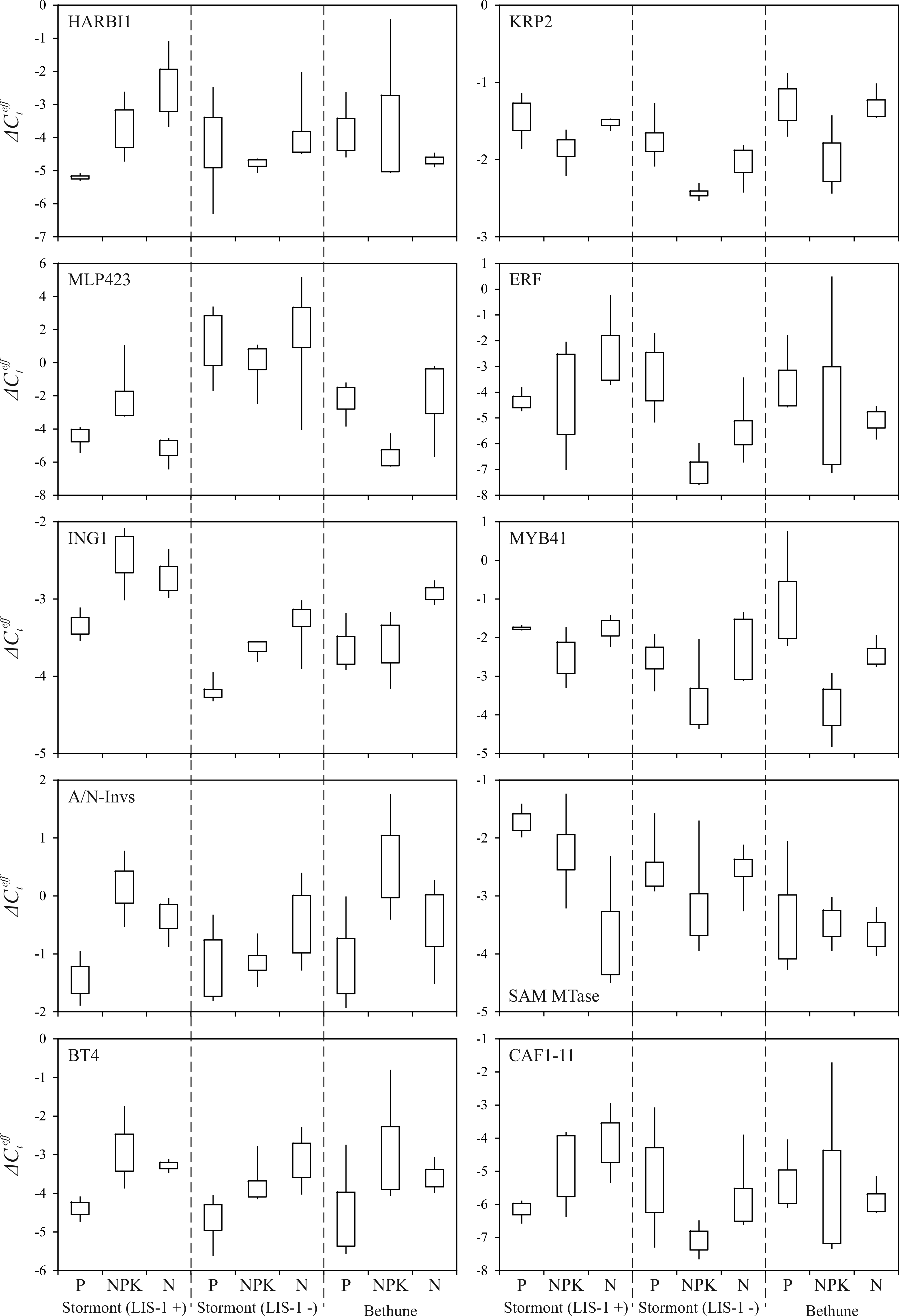

Supplement: Additional file 2: Figure S1. — Expression of ten genes in plants of cultivar ‘Bethune’ and line ‘Stormont Cirrus’ with (+) and without (-) LIS-1 grown under phosphate deficiency (P), excess nutrition (NPK), or normal (N) conditions. QPCR data. Rectangles correspond to the ranges containing 50 % of the values (between the 25th and 75th percentiles); the bars are the maximum and minimum ΔC t eff values. (TIF 873 kb) [file 12870_2016_927_MOESM2_ESM.tif]
